# Bumetanide for autism: more eye contact, less amygdala activation

**DOI:** 10.1038/s41598-018-21958-x

**Published:** 2018-02-26

**Authors:** Nouchine Hadjikhani, Jakob Åsberg Johnels, Amandine Lassalle, Nicole R. Zürcher, Loyse Hippolyte, Christopher Gillberg, Eric Lemonnier, Yehezkel Ben-Ari

**Affiliations:** 1 MGH/Martinos Center for Biomedical Imaging/ Harvard Medical School, Boston, USA; 2 Gillberg Neuropsychiatry Center, Gothenburg University, 41119 Gothenburg, Sweden; 3 Section for Speech and Language Pathology, Gothenburg University, 41119 Gothenburg, Sweden; 4 Service de Génétique Médicale, University of Lausanne, Lausanne, Switzerland; 5 Centre Hospitalier Universitaire, Limoges, France; 6 Neurochlore at Benari Institute of Neuroarcheology, Marseille, France

## Abstract

We recently showed that constraining eye contact leads to exaggerated increase of amygdala activation in autism. Here, in a proof of concept pilot study, we demonstrate that administration of bumetanide (a NKCC1 chloride importer antagonist that restores GABAergic inhibition) normalizes the level of amygdala activation during constrained eye contact with dynamic emotional face stimuli in autism. In addition, eye-tracking data reveal that bumetanide administration increases the time spent in spontaneous eye gaze during in a free-viewing mode of the same face stimuli. In keeping with clinical trials, our data support the Excitatory/Inhibitory dysfunction hypothesis in autism, and indicate that bumetanide may improve specific aspects of social processing in autism. Future double-blind placebo controlled studies with larger cohorts of participants will help clarify the mechanisms of bumetanide action in autism.

## Introduction

Eye-contact is experienced as stressful, or threatening in autism^1^, and the brain mechanisms underlying this reaction are beginning to be understood. We have recently shown that eye contact strongly activates the subcortical face-processing system, and in particular the amygdala, in participants with autism who are constrained to look in the eyes^2^. We also recently reported that individuals with autism exhibit more activation in the amygdala than individuals who do not have autism when constrained to look in the eyes of emotional faces expressing fear at low intensity^3^. These observations suggest that the amygdala might play an important role in the analysis and response of individuals with autism to emotive faces. It also raises the possibility that the amygdaloid network is overactive in individuals with autism notably when facing emotive stimuli.

**Figure 1:**
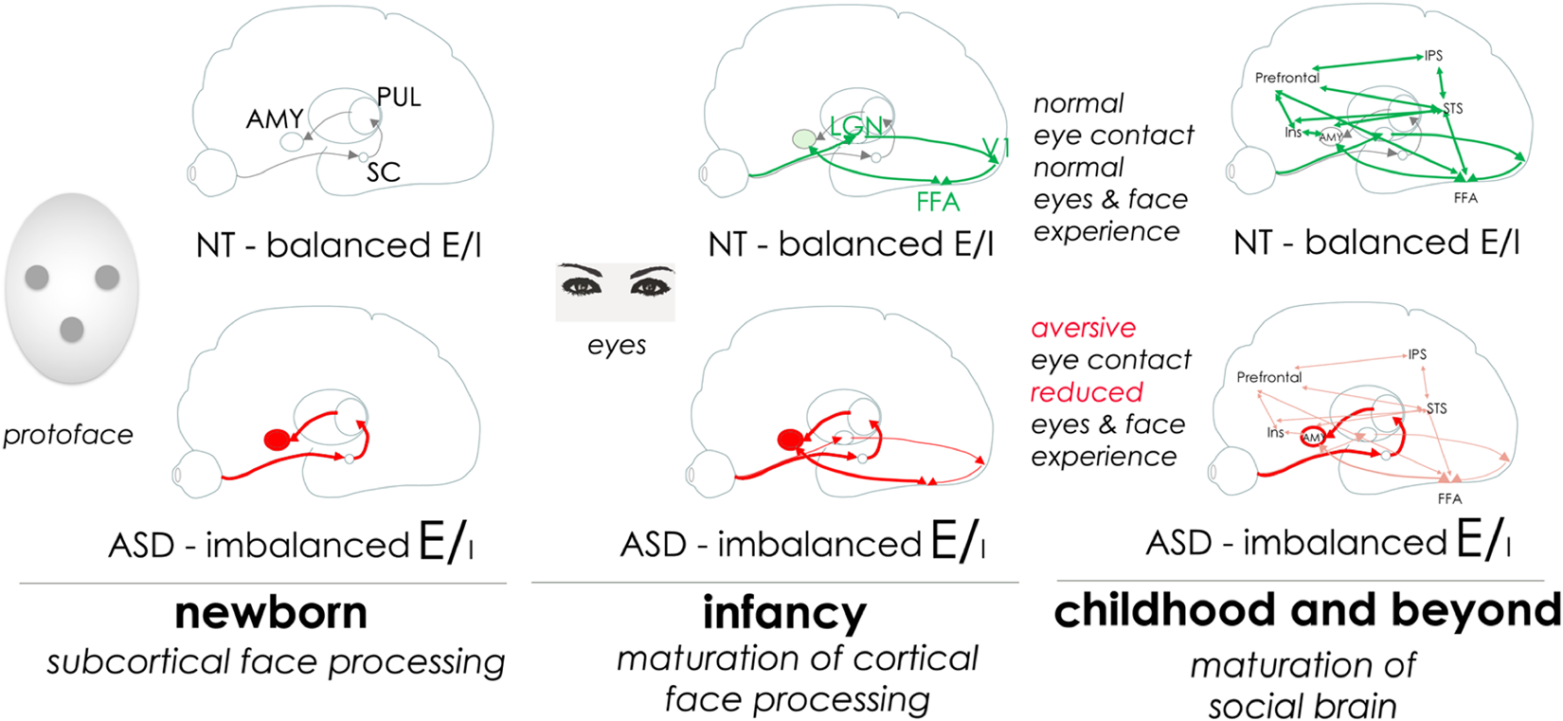
Working hypothesis: At birth, babies instinctively attend to ‘protofaces’, through the subcortical face processing system. Protofaces and faces activate the pathway going from SC to PUL and AMY. In autism (ASD), this pathway becomes over connected because of E/I imbalance. Later, eye contact activates the subcortical system, and helps normal maturation of the cortical face processing in neurotypicals (NT); but in ASD, eyes over-activate the system, in particular the amygdala, and provoke over-arousal, resulting in aversion to eye contact, and reduced experience with eyes and faces, which ultimately leads to an abnormal maturation of the “social brain”, (that includes e.g. the FFA, superior temporal sulcus (STS), intraparietal sulcus (IPS), prefrontal cortex and insula (INS)). – NT: neurotypicals; AMY: amygdala; PUL: thalamus pulvinar nucleus SC: superior colliculus; LGN: lateral geniculate nucleus; FFA: Fusiform Face Area; V1: primary visual cortex. E/I: excitatory/inhibitory.

The subcortical pathway is probably the substrate of the recently described preference of human fetuses for face-like protoface stimuli^4^, and of the newborns’ gazing preferences for protofaces^5–7^. This extraordinary sensitivity of newborns for faces helps the normal maturation of the visual cortical areas involved in face perception. Our working hypothesis in the present study is that in autism, already at early developmental stages, there is an imbalance favoring excitation in this system, that is persistently reinforced by the presence of the faces of caregivers. This, in turn, results in an abnormal, hyper-connected face-processing subcortical pathway, accompanied by a hypersensitivity of the amygdala to eye contact, leading to an aversive reaction to direct gaze, reduced eye contact and, as a result, atypical development of the social brain (Fig. 1).

Recent experimental investigations have provided an interesting conceptual framework and means of directly testing this hypothesis. Indeed, GABA – the principal inhibitory transmitter in the adult brain - depolarizes and excites immature neurons, stimulating neuronal growth and synapse formation. In a wide range of brain structures and animal species, there is a shift of the polarity of GABA actions due to a progressive reduction of the intracellular levels of chloride. Two chloride co-transporters – the bumetanide-sensitive chloride importer (NKCC1), and the chloride exporter (KCC2) - underlie this developmental sequence^8–10^, (for review, see^11^). In addition, in many neurological and psychiatric disorders, neurons have high (Cl^−^)_i_ and depolarizing/ excitatory actions of GABA^11^.

Experimental and clinical evidence suggests an excitatory to inhibitory (E/I) imbalance in autism^12^ (for review, see^13–15^), as well as in infantile epilepsy^16–19^ (for review, see^20^). Such imbalance is also supported by the paradoxical effects of benzodiazepinesin some cases of autism^21–23^ (for review on GABAergic system in ASD, see^24^), the alteration of gamma oscillations in autism, generated by GABAergic neurons^25–30^; and the reduced action of GABA in a paradigm of binocular rivalry^31^. Collectively these observations raise the possibility that high chloride levels and excitatory actions of GABA prevail in these brain regions leading to adverse response to eye contact in autism.

The highly specific NKCC1 chloride importer antagonist bumetanide that reduces efficiently (Cl^−^)_i_ levels^11,32^ can be used to test this hypothesis. Indeed, bumetanide has been shown to attenuate the severity of autism symptoms in children in two double blind randomized trials^33,34^ and in animal models of autism^35^. Also, we previously reported an improvement in emotional face processing both at the behavioral and at the brain activation level in a study where a group of adolescents and young adults with autism were treated for 10 month with bumetanide (^36^, see also ^37^). Specifically, we showed that after treatment, participants with autism were quicker and more accurate at recognizing emotions in a behavioral task, and that there was an increase in social brain activation in response to faces in an unconstrained stimulus presentation of dynamic faces.

In the present study, we examined the same group of individuals with autism (plus two additional participants) during the perception of dynamic emotional faces (same stimuli as described in^2,38^) at baseline and after 10 months of open-label bumetanide treatment, specifically examining the effect of bumetanide on amygdala activation. We previously reported the presence of an enhanced eye-contact effect in ASD, demonstrating that forcing them to look in the eyes significantly increased amygdala activation^2^. In the present study, we wanted to test the hypothesis that bumetanide treatment would reduce this eye-contact effect, and that this previously observed increase in amygdala activation during constrained eye contact condition would be reduced after bumetanide treatment. Because we wanted to show that this was not a scan/rescan effect, we also tested amygdala activation before and after treatment in a free viewing mode. Participants viewed two versions of the same short movies showing faces morphing from a neutral to an emotional expression. One version of these movies contained a red fixation cross in the region of the eyes that participants were asked to look at (CROSS condition), and the other was presented in free-viewing monde (NO CROSS condition). The order of the CROSS and NO-CROSS versions was counterbalanced across participants, so that about half of them saw the stimuli with NO-CROSS first. We tested the hypothesis that after bumetanide treatment, amygdala hyperactivation observed previously in the CROSS condition would normalize. In addition, we predicted that if there was less amygdala activation during eye contact, participants would increase their time spent looking in the eyes in a free-viewing paradigm after bumetanide treatment.

## Results

### fMRI

**Figure 2:**
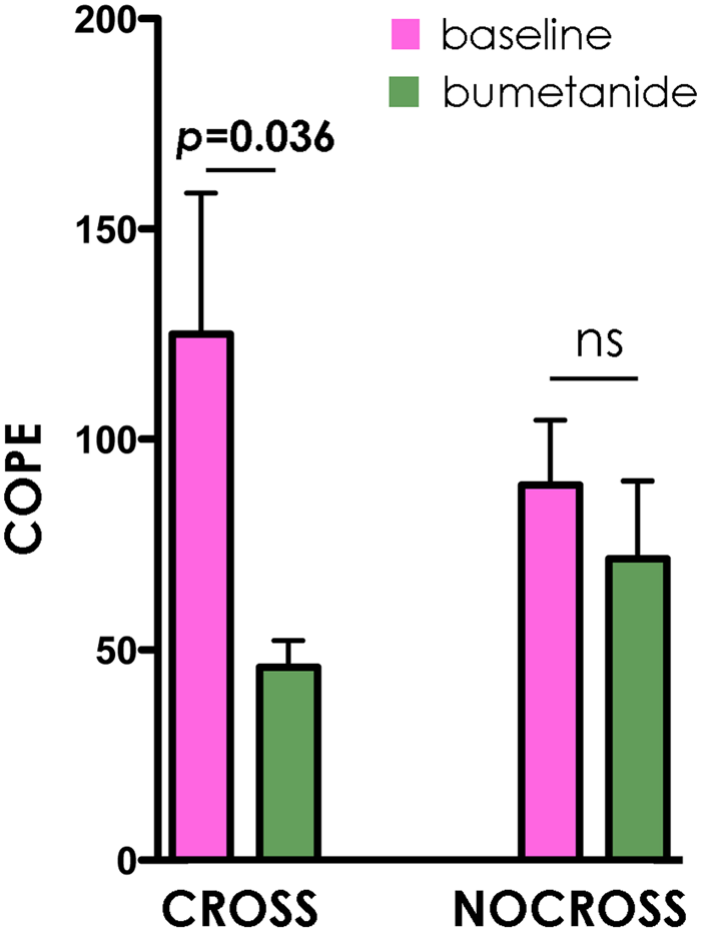
Descriptive plots of amygdala (left and right combined) activation at baseline (purple) and after 10 months of bumetanide treatment (green) in constrained eye contact (CROSS) and free viewing (NO CROSS) conditions. Data are shown as mean ± SEM.

Supporting our hypothesis, we found a significant decrease in amygdala activation compared with the baseline (pre-treatment) during constrained eye gaze (CROSS condition) following bumetanide treatment according to a nonparametric Wilcoxon signed ranks test (*z* = −2.100, *p* = 0.036, 2-tailed). These results were task-specific as no difference was seen in brain areas not involved in face-processing (e.g. the precuneus: z = 0.49, *p* = 0.64, frontal pole: z = −0.37, p = 0.71). In the amygdala, no difference from baseline to post treatment was seen in the free viewing-mode (NO CROSS) (*z* = −0.560, *p* = 0.57). These results suggest that the effect of bumetanide treatment might be specifically related to eye contact as hypothesized by our socio-affective extension of the E/I model. Also, the lack of change in the free viewing (NOCROSS) condition indicates that the findings are not driven by a general test-retest effect (Fig. 2).

Our working hypothesis concerned amygdala activation modulation by bumetanide. In order to examine whether these effects were specific only to the amygdala or extended to other brain regions, we also performed a whole brain analysis. This analysis indicated that the reduced activation of the amygdala during eye-contact by bumetanide (also present in whole brain analysis) was accompanied by reduction in brain areas involved in social, cognitive and somatic response. They included the lateral occipital cortex, the temporal pole, the insula, the cingulate, the parietal cortex and the premotor cortex. The primary visual cortex did not show any changes. See Supplementary material for Figure/Table S1.

### Eye-tracking

**Figure 3:**
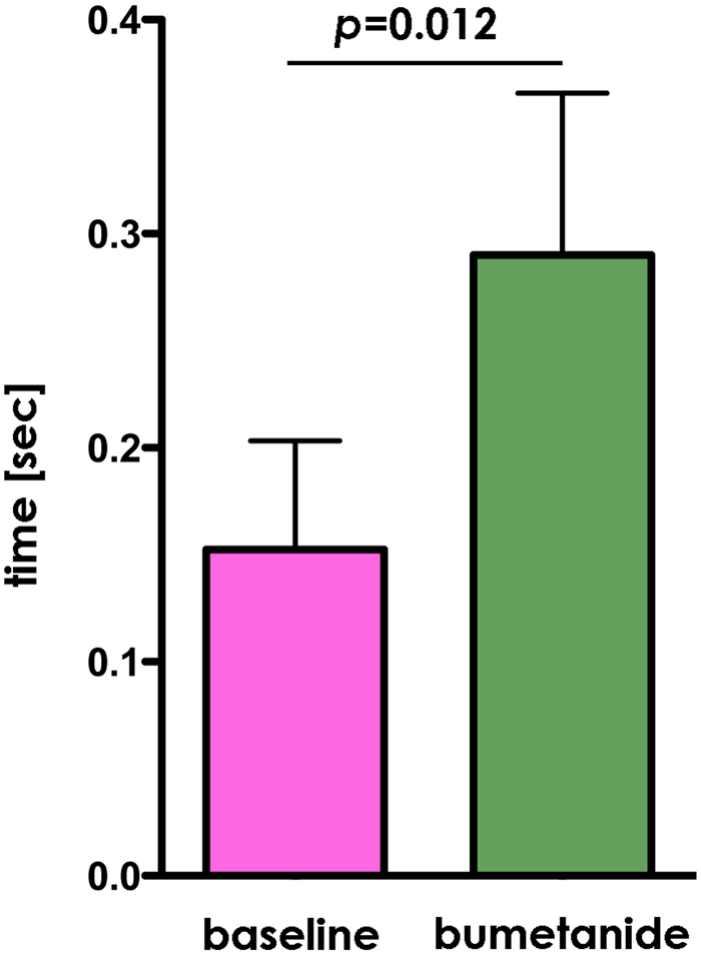
Descriptive plots of time spent in the eyes AOI at baseline (purple) and after bumetanide treatment (green) during free viewing showing that participants with ASD spontaneously spend more time looking at the eye region after treatment. Data are shown as mean ± SEM.

Eye tracking data were collected in the free-viewing mode. We examined the total time spent in an area of interest (AOI) that consisted of two ellipses over the left and the right eye. The Wilcoxon signed ranks tests revealed a significant increase in time looking in the eye AOI after treatment compared with baseline (*z = *2.521, *p* = 0.012, 2-tailed) (Fig. 3). A supplementary AOI revealed no significant change in time looking at the mouth treatment compared with baseline (*z* 1.260, *p* = 0.25, 2-tailed), confirming the specificity of this effect on eye contact.

## Discussion

Here we show that bumetanide treatment in autism normalizes amygdala activation in response to eye contact, and that it increases the time spontaneously spent in the eyes of dynamic emotional faces. These data complement our two previous studies that showed (1) increased activation in the social brain of participants with autism for emotional vs. neutral faces after bumetanide treatment in an unconstrained paradigms^36^, and (2) increase of subcortical face-processing network activation in autism compared to controls, including in the amygdala, when gaze is constrained in the eye region^2^. The novel results presented here are potentially of great theoretical and clinical importance.

The amygdala reacts strongly to eye contact^39^, even in people suffering from cortical blindness^40^. It is part of the subcortical pathway, a phylogenetically ancient brain structure^41,42^, also engaged in fast non-conscious threat perception^43,44^ that was recently objectively evidenced for the first time with intracranial electrophysiology in the human amygdala^45^. Here, we demonstrate that after 10 months of a treatment that putatively restores the E/I balance, the amount of amygdala activation in response to constrained eye contact in autism is significantly reduced. The decrease in amygdala activation is probably at the basis of the increased time spent spontaneously in the eyes after treatment, as observed here in our eye-tracking data, and of the improvement in emotional face processing as measured by a reduced reaction time and increased accuracy in an emotional matching task, as described in our first study^36^.

In addition to the reduction in amygdala activation, also visible in whole brain analysis, other areas involved in social, cognitive or somatic (preparation for action) responses also showed reduced activation after bumetanide, probably modulated by the amygdala reduction, as would be expected based on the data by^46^. Notably, these areas overlapped to a great extent with areas were we have previously demonstrated an eye-contact effect in typical individuals (see Fig. 1 in^38^). No changes were present in the primary visual cortex, excluding an effect due to arousal and attention alone. Although we postulate that the activation changes observed likely are the consequence of a modulation of the amygdala, we cannot exclude that the action of bumetanide may take place elsewhere, which is inherent to all experiments in which a drug is implemented *in vivo*.

The likelihood that these effects were due to a test-retest effect is small for the following several reasons: first, the paradigm used (in the initial study as well as in this one) was counterbalanced, with half of the participants seeing first the constrained condition, and the other half the free condition, yet no test-retest effect was noted within scanning session. More importantly, here we show that the treatment effect on amygdala activation is only present in the condition where gaze was constrained in the eye-region (the condition with a fixation cross, CROSS condition): during free-viewing (the condition without fixation cross, i.e. NO CROSS condition), there were no differences in amygdala activation between baseline and treatment.

The reduction of amygdala activation in response to eye contact may have important implications in the improvement of social emotional understanding in individuals with autism, as it may reduce the aversive effects of eye contact, and increase ‘face-time’ necessary for social learning and for the maturation of the social brain^47^. It is remarkable that we were able to see improvement both behaviourally and functionally in adolescents and young adults with autism^36^, suggesting that the window of opportunity to learn to decode emotions is not closed at that point of time. There is an interesting parallelism with experimental and clinical observations reported recently on the effects of Oxytocin on autism. Oxytocin has been associated with improvement in social competences in autism (for review, see^48^), tested in large clinical trials (for review, see^49^) and shown to reduce [Cl^−^]_i_^35,50,51^. The parallel actions of oxytocin and bumetanide stresses the important role [Cl^−^]_I_ levels in autism.

There are several important limitations to our study. First, given the small number of participants in this pilot study, these results will need replication in a larger group of individuals with autism, and with a larger age-range. Besides providing increased statistical power, a larger sample size would allow more sophisticated statistical approaches than the non-parametric technique used here, which was chosen given the small sample size. Indeed, with a larger sample size, we could directly test whether the increase in eye-gaze is mediated by the decrease in amygdala activation, as well as test interaction effects between experimental conditions. Hence this is an important avenue for future research. Second, this was an open-label treatment, with no placebo group, and future studies will need to re-examine these findings in a double-blind protocol, to exclude the possibility that this was an effect of neural adaptation, and/or a placebo effect. Third, we did not collect information about anxiety levels before and after treatment in all participants, and therefore could not measure whether reduction of amygdala activation was accompanied by a reduction in anxiety. Future studies should examine stress responses changes during eye contact after bumetanide treatment.

Our results demonstrate that bumetanide reduces threat response to eye contact and increase time spontaneously spent looking in the eyes, potentially allowing acquisition of the necessary information to improve social processing. Whether the actions of bumetanide are due to alterations of [Cl^−^]_I_ levels in amygdala neurons in patients with autism is beyond direct demonstration, but both the exquisite selectivity of the diuretic to the NKCC1 chloride importer and the converging experimental data are strongly suggestive that this is indeed the underlying mechanism.

## Methods

### Data availability statement

The datasets generated during and/or analyzed during the current study are available from the corresponding author on reasonable request.

### Ethics Statement

The study was approved by the Committee of Persons Protections CPP west 6-570-6/4/2009, and by the French Health Products Safety Agency (AFSAPS -A90936-66 4/12/2009). It was registered in ClinicalTrial.gov 3/1/2010 under number NCT01078714. The behavioral and fMRI protocols were approved by the Lausanne University Hospital Ethical Committee. Informed consent was obtained for all participants. All adult participants gave written consent before the start of the study. Minor participants gave their assent and one of their parents gave written consent. All procedures followed the Declaration of Helsinki.

Nine high-functioning individuals with autism (autistic disorder of Asperger syndrome) (1 female) took part in the present study. All participants underwent repeated scanning after 10 months of bumetanide treatment (1 mg/day). They were 21.4 ± 5.4 (mean ± SD) years old at the first testing session (range: 14.8-28.6).

Participants were diagnosed by an experienced clinician according to DSM IV-TR^52^ criteria and the Autism Diagnosis Observation Schedule (ADOS)^53^ and the Autism Diagnostic Interview-Revised (ADI-R)^54^. All participants met criteria for either autistic disorder (n = 3) or Asperger Syndrome (n = 6) based on their language development history. They were also asked to complete the Autism Quotient (AQ)^55^ (mean = 31.1 ± 5.4), Empathy Quotient (EQ)^56^ (mean = 19.9 ± 4.9) and other self-report questionnaires including the State-Trait Anxiety Inventory (n = 8) (STAI)^57^ (STAI trait: 56.1 ± 8.2; STAI state (48.9 ± 7.2). and the Toronto Alexithymia Scale (TAS)^58^ (n = 8) (57.6 ± 12.6). The performance IQ (PIQ) (105.6 ± 15.0) was assessed using Wechsler non-verbal scales^59,60^.

Participants always underwent fMRI session before the eye-tracking experiments.

### fMRI data acquisition

#### Experimental design

The same stimuli as those described for the eye-tracking (see details below) were used during the imaging experiment. Two versions of these movies were created, with one version containing a red fixation cross in the region of the eyes. In addition, a red fixation cross was present for 1 second between each movie at the same location, and periods of FIXATION were presented for 6 seconds at the beginning and at the end of each run, as well as for 3 seconds 7 times during each run interspersed between the blocks of emotional faces. Each participant viewed both versions (CROSS and NO-CROSS) during the scanning session (that also comported other tasks not reported in the present manuscript). The order of the CROSS and NO-CROSS versions was counterbalanced across participants, so that about half of them saw the stimuli with NO-CROSS first. Participants were instructed to carefully look at the videos and, in order to monitor their attention, to press a button every time they saw a blue cross between the stimuli, which happened 4 times during the CROSS and 4 times during the NO-CROSS condition. The stimuli presented during CROSS and NO-CROSS were identical, the only difference was the presence of a fixation cross during the CROSS version. Each block lasted 48 seconds. There were 8 blocks (2 for each emotion) and within one block, there were 8 stimuli (all different identities) for a total of 16 stimuli per condition.

#### Imaging data acquisition and analysis

Anatomical and functional MR images were collected in all participants with a 12-channel RF coil in a Siemens 3 T scanner (Siemens Tim Trio, Erlangen). T1-weighted anatomical images were acquired using an ME-MPRAGE (176 slices; 256 × 256 matrix; 1 × 1 × 1 mm voxels, echo time (TE): TE1: 1.64 ms, TE2: 3.5 ms, TE3: 5.36 ms, TE4: 7.22 ms; repetition time (TR): 2530 ms; flip angle 7°). Functional data were obtained using an echo planar imaging (EPI) sequence (47 AC-PC slices, 3 × 3 × 3 mm voxels, 64 × 64 matrix; FOV: 216; TE: 30 ms; TR: 3000 ms; flip angle 90°) lasting 384 seconds.

Functional MRI data processing, as well as preprocessing was carried out using FSL 5.0.2.2. Non-brain tissue was removed from high-resolution anatomical images using Christian Gaser’s VBM8 toolbox for SPM8^61^ and fed into feat. Data were motion-corrected using MCFLIRT and motion parameters were added as confound variables to the model. Residual outlier timepoints were identified using FSL’s motion outlier detection program and integrated as additional confound variables in the first-level General Linear Model (GLM) analysis. Preprocessing included spatial smoothing using a Gaussian kernel of 8 mm, grand-mean intensity normalization and highpass temporal filtering with sigma = 50.0 s.

In order to make sure that the results were not due to difference in data quality across sessions or conditions, we performed a 2 (Treatment: pre, post) by 2 (condition: CROSS, NOCROSS) repeated measure ANOVA on measures of relative motion during scanning. The interaction was not significant (*F*_1,7_ = 0.382, *p* = 0.556, *η*_*p*_^2^ = 0.052), indicating that differences were not due to issues related to differences in data quality.

Subject-level statistical analysis was carried out for each emotion in relation to fixation using FILM with local autocorrelation correction for both the CROSS and the NO-CROSS runs. Registration to high-resolution structural images was carried out using FLIRT. Registration to MNI standard space was then further refined using FNIRT nonlinear registration. Group-level analyses for each condition were performed using mixed effects GLM analysis using FLAME 1 + 2 with automatic outlier detection.

The region of interest (ROIs) consisted of the left and the right amygdala, anatomically defined from the Harvard-Oxford Subcortical atlas. We also examined two other ROIs, defined from the Harvard-Oxford Cortical atlas: the precuneus and the frontal pole. For each subject, the value of the maximum contrast of parameter estimate (COPE) was extracted for the left and the right amygdala, using the FSL Featquery tool in FSL.

Data from the first fMRI session were not obtained in one participant due to technical problems, and we are reporting results from the remaining eight.

In the analyses we computed average values for all emotional expressions across both the right and left amygdala at baseline and at post treatment in each of the condition (CROSS and NOCROSS). We tested the hypothesis that there would be a reduction of amygdala activation in the CROSS condition after treatment, but that treatment would not affect amygdala activation in the NO CROSS condition. Therefore comparisons from baseline to post treatment were carried separately for each condition out using Wilcoxon Signed ranks test.

### Eye-tracking

Data from one participant were not acquired in the eye-tracker at the second visit because of scheduling constraints. Gaze behavior was recorded using a Tobii T120 eye-tracking system and Tobii Studio 2.0.6. Participants sat comfortably around 65 cm in front of a 17” Tobii screen in a dimly lit room. The eye tracker consists of infrared light sources and cameras that are integrated into a thin film transistor technology (TFT) monitor. Using corneal reflection techniques, the eye tracker records the X and Y coordinates of participant’s eye position at 60 Hz. Using Tobii Studio (Tobii Technology, Sweden), a nine-point regular calibration was done prior to the experiment. Data was collected in real-time with an accuracy of 0.5 degrees. Eye fixations were determined (with Tobii Fixation Filter) using the criterion of eye position remaining within a 35 pixel area for a time greater than 80 ms. Data collection and analysis of areas of interest (AOI) data were determined with Tobii Studio 2.3.2.0.

Dynamic face stimuli were presented. We created a series of 32 short movies using faces from the NimStim database. Each of the eight identities chosen (four males) was used to generate four dynamic faces expressing different emotions. Each movie started with a neutral facial expression and ended with either a happy, angry, or fearful face. Morphs of neutral faces were also produced by creating a left-to-right morph between mirror images of neutral faces, in order to also have a dynamic component in this condition. The stimuli were presented in a pseudo-random order. Each movie lasted three seconds and was followed by a two-second picture of the final emotional expression. After each picture, a black-and-white marble rectangle was presented for 1.2, 1.5, or 1.8 seconds as an interstimulus interval. Both the stimuli and the interval were centered on the screen with dimensions of 10.6 cm × 13.1 cm. Participants were instructed to look carefully at the stimuli. We defined an AOI as two ellipses covering the left and the right eyes. Another control AOI consisted of an ellipse covering the mouth. Total fixation time to this region during the dynamic plus the still part of the stimuli was calculated using Tobii Studio 2.3.2.0.

We performed a 2-sided Wilcoxon Signed ranks test to compare gaze in the eyes AOI before and after treatment.

## Electronic supplementary material

Supplementary information
