# Supplementary material for: Bumetanide for autism: more eye contact, less amygdala activation
Source: Sci Rep. 2018 Feb 26;8:3602. doi: 10.1038/s41598-018-21958-x (PMC5827728; doi:10.1038/s41598-018-21958-x)
Supplement: Supplementary file 1 — Supplementary information [file 41598_2018_21958_MOESM1_ESM.docx]

**SREP-17-41794C**

**Supplementary material**

**Bumetanide for autism: more eye contact, less amygdala activation**

Nouchine Hadjikhani (MD, PhD)^1,2,*^, Jakob Åsberg Johnels (PhD)^2,3^, Amandine Lassalle (PhD)^1^, Nicole R Zürcher (PhD)^1^, Loyse Hippolyte (PhD)^4^, Christopher Gillberg (MD, PhD)^2^, Eric Lemonnier (MD, PhD)^5^, Yehezkel Ben-Ari (PhD)^6^.

^1^ MGH/ Martinos Center for Biomedical Imaging/ Harvard Medical School, Boston, USA

^2^ Gillberg Neuropsychiatry Center, Gothenburg University, 41119 Gothenburg, Sweden

^3^ Section for Speech and Language Pathology, Gothenburg University, 41119 Gothenburg, Sweden

^4^ Service de Génétique Médicale, University of Lausanne, Lausanne, Switzerland

^5^ Centre Hospitalier Universitaire, Limoges, France

^6^ Neurochlore, Marseille, France

*Correspondence to: [nouchine@nmr.mgh.harvard.edu](mailto:nouchine@nmr.mgh.harvard.edu)

**Supplementary material**


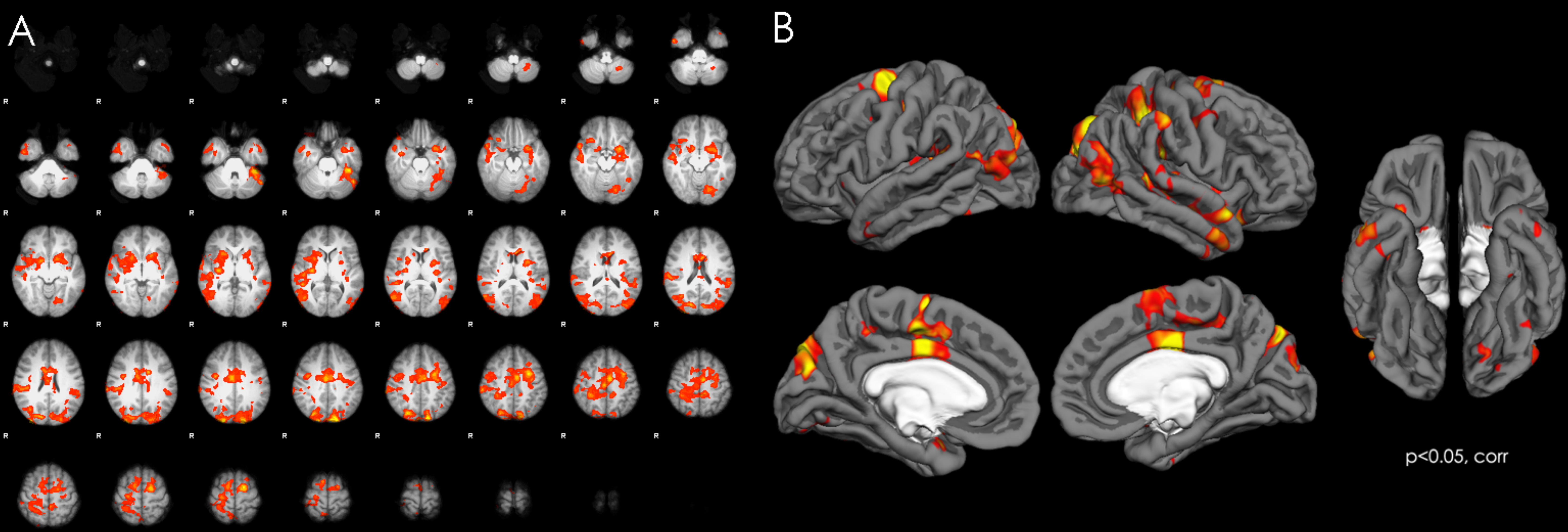


**Figure S1:** Whole brain analysis of areas showing reduced activation to constrained eye-contact after bumetanide treatment. Panel A: volume data, p<0.05, cluster corrected z=2.3; Panel B: data presented on the surface.

| **Area** | **side** | **Z-value** | **X** | **Y** | **Z** |
| --- | --- | --- | --- | --- | --- |
| lateral occipital cortex, inferior | R | 4.85 | 53 | -68 | 8 |
|  | L | 4.55 | -54 | -76 | 10 |
| lateral occipital cortex, sup | R | 5.71 | 26 | -76 | 40 |
|  | L | 5.91 | -14 | -86 | 36 |
| precuneus | R | 4.46 | -4 | -42 | 52 |
|  | L | 4.07 | -8 | -74 | 44 |
| occipital fusiform gyrys | L | 4.59 | -18 | -76 | -12 |
| intraparietal sulcus | R | 4.75 | 30 | -82 | 28 |
|  | L | 4.45 | -24 | -86 | 26 |
| superior parietal lobule | R | 4.24 | 32 | -44 | 62 |
| Post. superior temporal gyus | R | 4.09 | 66 | -30 | 14 |
| posterior supramarginal gyrus | L | 4.54 | -56 | -42 | 18 |
| orbitofrontal cortex | R | 4.33 | 28 | 22 | -18 |
| temporal pole | R | 5.01 | 54 | 10 | -18 |
|  | L | 4.93 | -30 | 4 | -24 |
| precentral gyrus | R | 4.96 | 26 | -22 | 62 |
|  | L | 4.49 | -32 | -6 | 48 |
| insula | R | 3.96 | 38 | 4 | -6 |
|  | L | 3.30 | -34 | -20 | 10 |
| putamen | R | 4.50 | 24 | 16 | 0 |
|  | L | 4.69 | -16 | 8 | 10 |
| amygdala | R | 3.80 | 28 | 2 | -22 |
|  | L | 4.49 | -26 | 0 | -16 |
| anterior cingulate | R | 5.32 | 4 | -6 | 36 |
|  | L | 5.19 | -6 | -6 | 42 |
| supplementary motor area | L | 4.93 | -6 | -8 | 52 |
|  | R | 3.81 | 10 | -8 | 54 |
| Paracingulate gyrus | R | 3.99 | 6 | 10 | 44 |
| cerebellum VI | L | 5.05 | -36 | -42 | -32 |
| cerebellum VIIIa | L | 4.38 | -24 | -54 | -48 |

**Table S1 –** areas showing reduced activation during constrained eye-contact after bumetanide treatment, p<0.05, cluster corrected.
